# Upcycling Polypropylene Waste into Carbon Anode Materials for Lithium-Ion Batteries

**DOI:** 10.3390/ma19194153

**Published:** 2026-09-29

**Authors:** Yryszhan Tashenova, Kuanysh Kurtibay, Dossym Yeskozha, Aliya Mukanova, Sung-Soo Kim, Arailym Nurpeissova

**Affiliations:** 1 Institute of Batteries (IoB), Kabanbay Batyr Ave. 53, Astana 010000, Kazakhstan; iriszhan9696@gmail.com (Y.T.); kurtibayqb@gmail.com (K.K.); dos.eskozha@gmail.com (D.Y.); aliya.mukanova@nu.edu.kz (A.M.); 2 Institute of New Materials and Energy Technologies, Kabanbay Batyr Ave. 53, Astana 010000, Kazakhstan; 3 Graduate School of Energy Science and Technology, Chungnam National University, 99 Daehak-ro, Yuseong-gu, Daejeon 34134, Republic of Korea

**Keywords:** polypropylene waste, upcycling, sulfonation cross-linking, hard carbon, lithium-ion battery anode

## Abstract

The growing accumulation of plastic waste is a serious environmental problem, and converting this waste into useful materials is an attractive solution. In this work, waste polypropylene (PP) was converted into hard carbon anode materials for lithium-ion batteries. When heated, PP decomposes into volatile hydrocarbons and leaves almost no solid residue, so it cannot be carbonized on its own. The waste PP was therefore first treated with concentrated sulfuric acid, which cross-links the polymer chains into a rigid, infusible network. This suppresses volatilization and promotes the formation of a thermally stable carbonaceous residue. The cross-linked material was then carbonized under argon at 700, 800, 900 and 1000 °C, and 22 to 24% of the original PP mass was recovered as carbon powder, depending on the carbonization temperature. X-ray photoelectron spectroscopy showed that the carbon surfaces retain 6.3–7.5 at% oxygen but almost no sulfur (≤0.2 at%), so sulfur is essentially absent from the final carbons. The carbonization temperature influenced the structure of the carbon and its performance in a battery. Among all samples, PP-900 delivered the highest reversible capacity at 0.1 A g^−1^, reaching 373.1 mAh g^−1^ at the 100th cycle. The first-cycle Coulombic efficiency was low for all samples (30–47%) and represents the main limitation of these materials. These results show that acid cross-linking followed by carbonization is a feasible route for converting PP waste into carbon anode material, and identify the low first-cycle efficiency and the rate performance at high current density as the principal issues to be addressed in further work.

## 1. Introduction

The rapid growth of global plastic waste has become a major environmental challenge, creating an urgent need for sustainable management strategies. One promising approach is the conversion of waste plastics into functional carbon materials for energy storage applications [1,2]. This strategy not only reduces environmental burdens but also has been proposed as a potentially cost-effective route to carbon-based electrodes for lithium-ion battery (LIB) systems [2]. Given that global plastic production exceeds 400 million tones annually, shifting from a linear to a circular economy is increasingly critical [3].

Among commodity plastics, polypropylene (PP) is of particular interest due to its widespread use, low density, chemical resistance, and low cost. PP is extensively applied in packaging, automotive components, medical devices, and textiles, making it a significant contributor to plastic waste streams [4,5]. However, PP is a non-carbonizable polymer with a saturated hydrocarbon backbone, which results in low carbon yield and poor structural stability during direct thermal treatment. Its thermal degradation is dominated by chain scission and the release of volatile hydrocarbons, limiting the formation of stable carbon frameworks [6,7].

Despite these limitations, PP can form carbon-rich structures, including aromatic carbon domains, when appropriate stabilization is applied prior to carbonization [8]. These characteristics have driven increasing interest in converting PP waste into carbonaceous materials for electrochemical energy storage. With proper processing control and material design, waste-derived PP carbons offer a pathway to combine plastic waste reduction with the development of sustainable electrode materials for batteries [2].

Sulfuric-acid-induced sulfonation and cross-linking of polyolefins, followed by carbonization, is an established route to non-graphitizable carbons and has already been applied to PP-based precursors. Lee et al. converted PP disposable masks into hard carbons for sodium-ion anodes and showed that PP remains non-graphitizable even after heat treatment at 2400 °C [9]. Wang et al. used sulfonation-induced cross-linking of recycled PP separators to obtain structure-tailored hard carbons for sodium storage [10], and Chae et al. compared sulfonation, nitration and thermal oxidation as stabilization routes for PP mask waste in lithium-ion anodes [8]. Building on these studies, the present work fixes a single sulfonation protocol and varies the carbonization temperature between 700 and 1000 °C, so that the structural changes produced by temperature alone, namely interlayer spacing, defect density, aromatic cluster growth and pore texture, can be mapped onto lithium storage behaviour for one feedstock. That feedstock is post-consumer rigid PP packaging, which differs in processing history and additive content from the melt-blown fibre and separator films used previously. The resulting temperature-resolved structure–property correlation identifies the first-cycle irreversible loss, rather than the reversible capacity, as the property that limits this class of material.

## 2. Materials and Methods

### 2.1. Materials

Waste PP was sourced from commercially available containers and cups obtained from local supermarkets. Prior to use, the collected PP materials were manually cut into small pieces, followed by thorough washing with deionized water and ethanol to remove impurities. Sulfuric acid (98.0%, Sigma-Aldrich, St. Louis, MO, USA) was used for the pre-treatment of the PP waste. High-purity argon gas was utilized to maintain an inert atmosphere during the carbonization process.

For electrochemical half-cell assembly, lithium metal foil was obtained from Alfa Aesar, Haverhill, MA, USA. A Celgard 2400 PP separator was supplied by Celgard LLC, Charlotte, NC, USA. The electrolyte consisted of 1 M lithium hexafluorophosphate (LiPF_6_) in a 1:1:1 (*v*/*v*/*v*) mixture of ethylene carbonate (EC), dimethyl carbonate (DMC) and diethyl carbonate (DEC), all procured from Sigma-Aldrich. Polyvinylidene fluoride (PVDF) served as the binder, and conductive carbon black (Super P, Timcal, Bodio, Switzerland) was used as the conductive additive for electrode fabrication. N-methyl-2-pyrrolidone (NMP) from Sigma-Aldrich was used as the solvent for slurry preparation.

### 2.2. Materials Synthesis

For sulfonation, approximately 5 g of the shredded PP pieces were immersed in 50 mL of concentrated sulfuric acid in a reaction vessel, which was placed on a hot plate set to 180 °C and held for 6 h. After sulfonation, the samples were washed thoroughly with deionized water in a deep beaker until the rinse water reached neutral pH in order to remove residual acid, the water being decanted through a sieve that retained the plastic pieces, and were then dried in a vacuum oven at 60 °C for 12 h.

The dried sulfonated samples were subsequently carbonized in a single-zone horizontal tube furnace (Bes Saiman Group, Almaty, Kazakhstan) under a continuous argon flow at temperatures between 700 and 1000 °C using a heating rate of 5 °C min^−1^. Upon reaching the target temperature, the samples were held for 2 h before cooling naturally to room temperature under argon. The carbonized products obtained at 700, 800, 900 and 1000 °C were denoted PP-700, PP-800, PP-900 and PP-1000, respectively. The whole preparation was carried out in triplicate. For each replicate set, approximately 20 g of pristine PP was divided into four portions of about 5 g, and every portion was sulfonated independently and then carbonized at one of the four temperatures, so that three independent syntheses were obtained for each sample, for twelve experiments in total. The masses of the pristine PP, of the washed and vacuum-dried sulfonated precursor and of the recovered carbon powder were recorded for every experiment; the individual values are listed in Table S1 and the derived yields are summarized in Table S2. Sulfonation increased the mass of the polymer by 2.7 ± 1.0 wt% (n = 12). This value understates the mass added by the acid treatment, because a small fraction of the sulfonated pieces was lost mechanically when the wash water was decanted through the sieve. The loss varies from batch to batch, which accounts for the comparatively large scatter of the mass gain in Table S1 against the much tighter reproducibility of the yields themselves. Two yields are therefore distinguished. Referred to the dried sulfonated precursor, the carbonization yield decreased from 23.5 ± 0.4 wt% at 700 °C to 20.9 ± 0.8 wt% at 1000 °C; referred to the pristine PP charged, the overall yield decreased from 24.3 ± 0.1 to 21.6 ± 0.8 wt% over the same range (mean ± standard deviation, n = 3). All yields refer to the powder recovered before ball milling. Because the pristine PP charged includes the pieces subsequently lost during washing, the overall yield describes the process as operated, handling losses included, and is therefore a lower bound on the conversion of PP into carbon; the carbonization yield is not affected, since it refers to the precursor mass actually charged into the furnace. The resulting carbon materials were then pulverized using a planetary ball mill (PULVERISETTE 7, Fritsch, Idar-Oberstein, Germany) at 450 rpm for 4 h to obtain fine powders for subsequent characterization and electrochemical evaluation.

### 2.3. Materials Characterization

The surface morphology of the carbonized samples was investigated using scanning electron microscopy (SEM, Zeiss Crossbeam 540, Carl Zeiss AG, Oberkochen, Germany). Energy-dispersive X-ray spectroscopy (EDS, JEOL JSM-IT800, JEOL Ltd., Tokyo, Japan) elemental mapping of carbon, oxygen and sulfur was carried out on the same microscope, and the composition was quantified from the map sum spectrum of each mapped area. The nanoscale structure was further examined by transmission electron microscopy (TEM, JEOL JEM-1400PLUS, JEOL Ltd., Tokyo, Japan). Raman spectra were recorded on a Horiba LabRam Evolution spectrometer (Horiba Scientific, Kyoto, Japan) under 532 nm excitation at a laser power of 1 mW, using an exposure time of 10 s and 10 accumulations per spectrum, and covering the range 500–3200 cm^−1^. After subtraction of a linear baseline, the D band near 1350 cm^−1^ and the G band near 1580 cm^−1^ were resolved by fitting pseudo-Voigt profiles in OriginPro 2024 (10.1). The I_D_/I_G_ ratio was calculated from the heights of the fitted bands, that is, from their maximum intensities above the subtracted baseline, and used to assess the degree of structural disorder of the carbons. The crystallinity and phase composition of the carbon materials were determined by X-ray diffraction (XRD, MiniFlex Rigaku, Tokyo, Japan). The specific surface area and porosity of the carbonized samples were evaluated by nitrogen physisorption at 77 K using a Micromeritics 3Flex analyzer (Micromeritics Instrument Corporation, Norcross, GA, USA). Specific surface areas were obtained by the BET method and pore size distributions by DFT analysis. Chemical changes induced by the acid treatment were followed by Fourier-transform infrared spectroscopy (FTIR, Nicolet i10, Thermofisher, Waltham, MA, USA). Thermogravimetric analysis (TGA) was performed using a simultaneous thermal analyzer (STA 449 F5 Jupiter, NETZSCH-Gerätebau GmbH, Selb, Germany) to evaluate the thermal stability of the samples. Measurements were conducted under a continuous argon flow by heating the samples from 30 to 1000 °C at a rate of 10 °C min^−1^. The evolved gases were analyzed simultaneously by a quadrupole mass spectrometer coupled to the thermal analyzer (TGA-MS), with the ion currents of selected mass-to-charge fragments recorded as a function of temperature. The surface composition and chemical states of the carbonized samples were determined by X-ray photoelectron spectroscopy (XPS, NEXSA, Thermo Scientific, West Sussex, UK). Survey spectra and high-resolution C 1s, O 1s and S 2p spectra were recorded, the binding energy scale was referenced to the main C 1s line at 284.8 eV, and peak fitting and quantification were carried out in the Avantage software (5.9931.0.6755).

### 2.4. Electrochemical Characterization

To evaluate the electrochemical performance of the synthesized carbon materials, CR2032 coin-type half-cells were assembled, using the synthesized carbon powders as the anode active material. The electrode slurry was prepared by mixing the active material, conductive carbon black (Super P) and PVDF in NMP at a weight ratio of 8:1:1. The mixture was thoroughly homogenized using a planetary centrifugal vacuum mixer (Thinky Mixer ARV-310, Thinky Corporation, Tokyo, Japan) to obtain a uniform slurry. The slurry was then coated onto copper foil using a doctor blade and subsequently dried under vacuum at 80 °C for 12 h to remove the solvent. Circular electrodes with a diameter of 14 mm were punched from the dried foil, yielding an active material mass loading of approximately 1.2–1.5 mg cm^−2^.

The coin cells were assembled in an argon-filled MBraun glove box with oxygen and moisture levels maintained below 0.1 ppm. The fabricated carbon electrodes served as the working electrode, while lithium metal foil (Alfa Aesar, 99.9%) was used as both the counter and the reference electrode. The two electrodes were separated by a Celgard 2400 separatorsoaked in the electrolyte described in Section 2.1. Galvanostatic charge–discharge measurements were carried out at current densities between 0.1 and 2 A g^−1^ using a Neware battery testing system (Neware, Shenzhen, China). Cyclic voltammetry (CV) was performed on a BioLogic electrochemical workstation (BioLogic, Seyssinet-Pariset, France) at a scan rate of 0.1 mV s^−1^ over a potential window of 0.01–3.0 V (vs. Li^+^/Li). Electrochemical impedance spectroscopy (EIS) measurements were conducted using the same instrument, applying a 5 mV sinusoidal perturbation across a frequency range of 100 kHz to 0.01 Hz. For every sample, two to three cells were assembled and measured independently, and the cycling and rate capability tests were run on separate cells. All curves shown are from a representative cell of each set, and the capacities quoted in the text and tables are those of that cell.

## 3. Results

### 3.1. Material Characterization

As PP predominantly decomposes into volatile products during pyrolysis, a pre-treatment step is required to enable carbon residue formation. Sulfonation using sulfuric acid provides an effective chemical route by transforming thermally unstable linear PP chains into a stabilized structure capable of surviving subsequent high-temperature carbonization [11,12].

**Figure 1:**
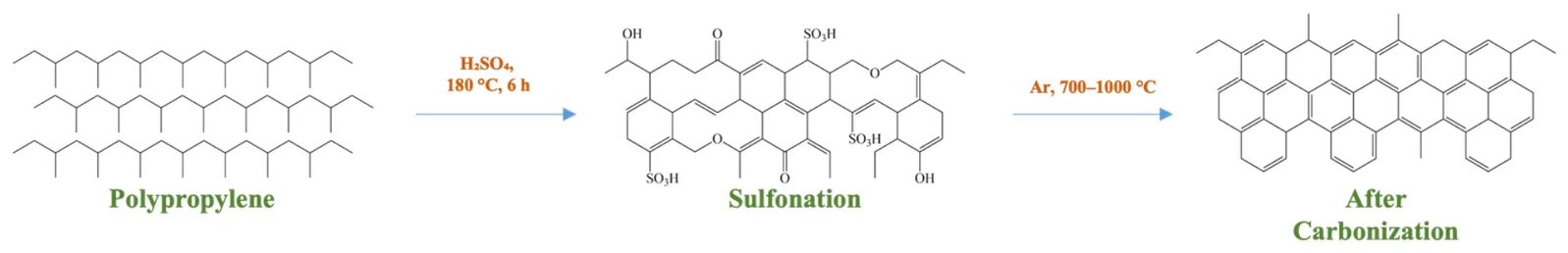
Mechanism proposed in the literature for carbon formation from waste PP through H_2_SO_4_-induced cross-linking (sulfonation) followed by high-temperature carbonization under argon atmosphere [8].

A reaction pathway consistent with previous reports on polyolefin sulfonation can be proposed. This mechanism is inferred from the available literature and supported by the observed spectroscopic changes. The sequence is thought to begin with electrophilic addition of sulfur trioxide or sulfuric acid species at secondary and tertiary carbon atoms of the PP backbone, which disrupts the initial crystalline order of the polymer. As the treatment proceeds, the sulfonated groups are proposed to undergo radical-mediated transformations, in which hydrogen abstraction and homolytic cleavage of hydroxy sulfonyl radicals generate carbon–carbon double bonds along the polymer chains, with H_2_O and SO_2_ released as byproducts [8,9,10,11]. These unsaturated sites would then promote intermolecular coupling between adjacent chains, forming covalent cross-links and giving a rigid, infusible three-dimensional network. This structural transformation prevents the melting and volatilization that typically occur for untreated PP, allowing the material to retain its structure during thermal processing and form a carbonaceous residue. Upon subsequent carbonization under argon, this cross-linked network undergoes progressive aromatization, in which aliphatic segments reorganize into small polyaromatic clusters. This reorganization is generally reported to be accompanied by the release of gaseous species such as H_2_O, CO_2_, CO and SO_2_, arising from decomposition of residual oxygen- and sulfur-containing groups [9,11,12]. In the present TGA-MS experiments, however, the CO_2_ and SO_2_ channels showed no distinct evolution above the instrument background. A schematic representation of the sulfonation-induced stabilization and subsequent carbonization process is shown in Figure 1.

This reorganization produces a disordered carbon framework containing both amorphous and graphitic domains, which is generally considered beneficial for lithium-ion storage on account of the increased number of defect sites and the expanded interlayer spacing [9,13]. The effectiveness of this stabilization is reflected in the significantly higher carbon yield of the treated precursor compared to pristine PP, which otherwise fully volatilizes during heating.

**Figure 2:**
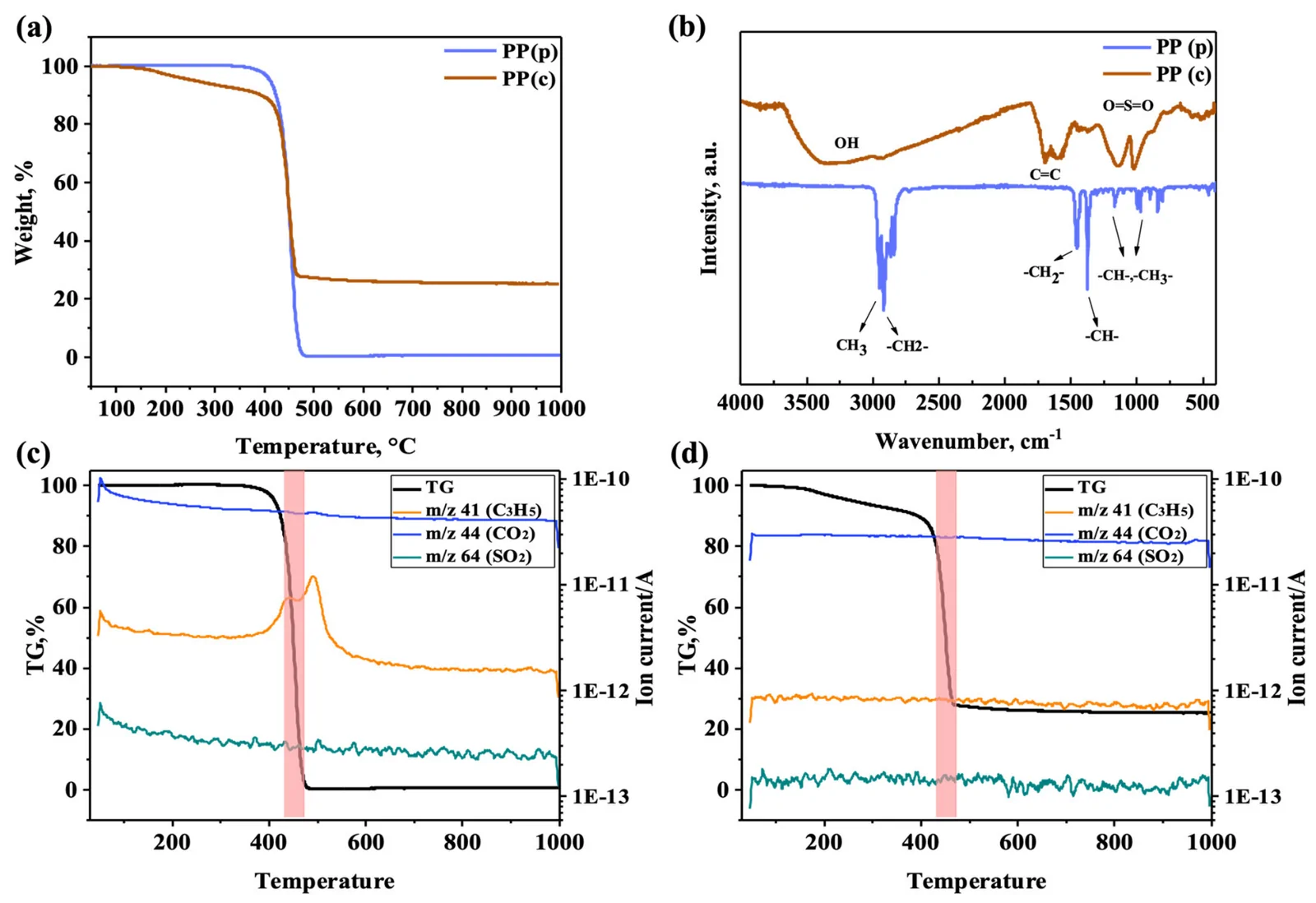
Thermal and structural characterization of pristine (PP(p)) and cross-linked (PP(c)) polypropylene by (**a**) TGA, (**b**) FTIR, and (**c**,**d**) TGA-MS evolved-gas analysis of (**c**) pristine and (**d**) cross-linked PP.

The thermogravimetric analysis (TGA) curves in Figure 2a demonstrate a pronounced change in thermal stability following sulfuric acid pre-treatment.

Pristine PP (PP(p)) exhibits a single-step degradation behaviour, with rapid decomposition initiating at approximately 400 °C and reaching a maximum weight loss rate near 448 °C. By 500 °C, almost no residual mass remains, confirming complete volatilization of the linear polymer chains into low-molecular-weight hydrocarbons [6,7]. This behaviour confirms that untreated PP cannot be directly converted into a carbonaceous residue.

In contrast, the sulfonated and cross-linked PP (PP(c)) shows a distinctly altered degradation pathway. An initial weight loss observed between 150 and 200 °C is assigned to the release of physiosorbed water and to the decomposition of labile sulfonyl and hydroxyl functional groups introduced during the acid treatment. FTIR confirms that sulfonyl and hydroxyl groups are present in the cross-linked precursor (Figure 2b), but does not establish that these are the species released in this temperature interval. The main decomposition step around 450 °C is significantly suppressed compared with PP(p), indicating enhanced thermal stability. Importantly, the material retains a stable residue of approximately 25 wt% at 1000 °C, expressed relative to the mass of the sulfonated precursor placed in the thermobalance, which reflects the formation of an infusible cross-linked network that resists complete volatilization [8,9]. This residue is an upper bound on the isolated yield rather than the yield itself, because the thermobalance ramps through the final temperature whereas the tube furnace holds it for 2 h. The measured carbonization yields are accordingly 2 to 4 wt% lower in absolute terms (Table S2).

Fourier-transform infrared (FTIR) spectroscopy (Figure 2b) further confirms the chemical transformations responsible for this stabilization. Pristine PP(p) displays characteristic aliphatic C-H stretching vibrations at 2970, 2915, and 2850 cm^−1^, along with bending modes at 1456 and 1373 cm^−1^, consistent with a saturated hydrocarbon backbone. After sulfonation, the spectrum changes significantly. A broad band centred around 3400 cm^−1^ indicates the introduction of hydroxyl-containing species, while a new absorption appears at approximately 1650 cm^−1^. This position is not diagnostic on its own: C=C stretching of newly formed unsaturation, the H-O-H bending mode of adsorbed water and conjugated carbonyl groups produced by partial oxidation all absorb between about 1620 and 1680 cm^−1^. Given the intensity of the hydroxyl band at 3400 cm^−1^, a contribution from adsorbed water cannot be excluded, and the 1650 cm^−1^ feature is therefore regarded as consistent with, but not as proof of, early-stage aromatization. In addition, new peaks at 1150–1200 cm^−1^ and 1040 cm^−1^ correspond to asymmetric and symmetric stretching of sulfonyl (O=S=O) groups bonded to the polymer backbone. Concurrently, the pronounced weakening of the original C-H vibrations indicates substantial disruption of the linear PP structure, consistent with the formation of a cross-linked network [11,12]. The evidence for the structural transformation consequently rests on the combination of these changes and on the char yield measured by TGA, rather than on the 1650 cm^−1^ band alone.

Overall, the combined TGA and FTIR results demonstrate that sulfuric acid treatment effectively transforms PP from a thermally unstable linear polymer into a stabilized, cross-linked structure. This chemical modification introduces functional groups that promote radical-mediated cross-linking and olefination, enabling the formation of an infusible network that resists complete thermal degradation. As a result, the treated material yields a stable carbon residue during high-temperature pyrolysis, whereas pristine PP undergoes full volatilization without carbon formation [6,7].

To identify the volatile species responsible for the mass losses recorded in the TGA curves, the thermal decomposition of pristine and cross-linked PP was simultaneously monitored by coupled thermogravimetry–mass spectrometry (TGA-MS). During heating under argon, the gases evolved from the sample were transferred directly to the mass spectrometer, and the ion currents of selected mass-to-charge (*m*/*z*) fragments were recorded as a function of temperature. The signals of the argon carrier gas (*m*/*z* 36, 38 and 40) and the water background were excluded, and the channels presented in Figure 2c,d are *m*/*z* 41 (the C_3_H_5_^+^ hydrocarbon fragment), 44 (CO_2_) and 64 (SO_2_).

For pristine PP (Figure 2c), a single, well-defined evolution of the C_3_ hydrocarbon fragment (*m*/*z* 41, C_3_H_5_^+^) is observed, rising sharply between ~430 and 500 °C and reaching a maximum near 490 °C, coincident with the single mass-loss step of the TG curve. No corresponding features appear in the oxygen- or sulfur-containing channels. This confirms that untreated PP decomposes through random chain scission of its saturated backbone into low-molecular-weight volatile hydrocarbons, which are released almost quantitatively and leave essentially no solid residue (<1%) [6,7,14].

For the cross-linked PP (Figure 2d), the hydrocarbon evolution is markedly suppressed. The *m*/*z* 41 ion current is substantially lower than for pristine PP and no longer shows a distinct release peak at the main mass-loss step. This indicates that the sulfonation-induced cross-linked network inhibits fragmentation into volatile hydrocarbons, so that approximately 25 wt% of the precursor mass is retained as a solid carbon residue rather than escaping as gas [8,9,10]. The CO_2_ (*m*/*z* 44) and SO_2_ (*m*/*z* 64) channels remained close to the instrument background in both samples, without a distinct evolution step under these conditions. The EDS results presented (Figure S2) show that the carbonized samples contain almost no sulfur, so the final carbons are essentially sulfur-free. These measurements establish the low sulfur content of the final carbons but do not determine the pathway of sulfur removal. Sulfur may have been removed during both the washing step following sulfonation and the subsequent carbonization. During washing, residual and physisorbed sulfuric acid may be removed, while during carbonization, sulfur may be released as unmonitored species such as H_2_S, COS, or CS_2_ [15,16], or gradually as SO_2_ below the detection limit of the instrument.

Taken together, the TGA-MS data provide molecular-level evidence for the suppression of hydrocarbon volatilization that underlies the stabilization concept inferred from the TGA and FTIR results. Whereas pristine PP is converted almost entirely into volatile C_3_ hydrocarbons and leaves no carbon, cross-linking suppresses this volatilization and preserves an infusible carbon framework. This accounts for the pronounced difference in char yield between the two materials and supports sulfuric acid pre-treatment as an effective route for enabling carbon formation from PP waste [10,11].

**Figure 3:**
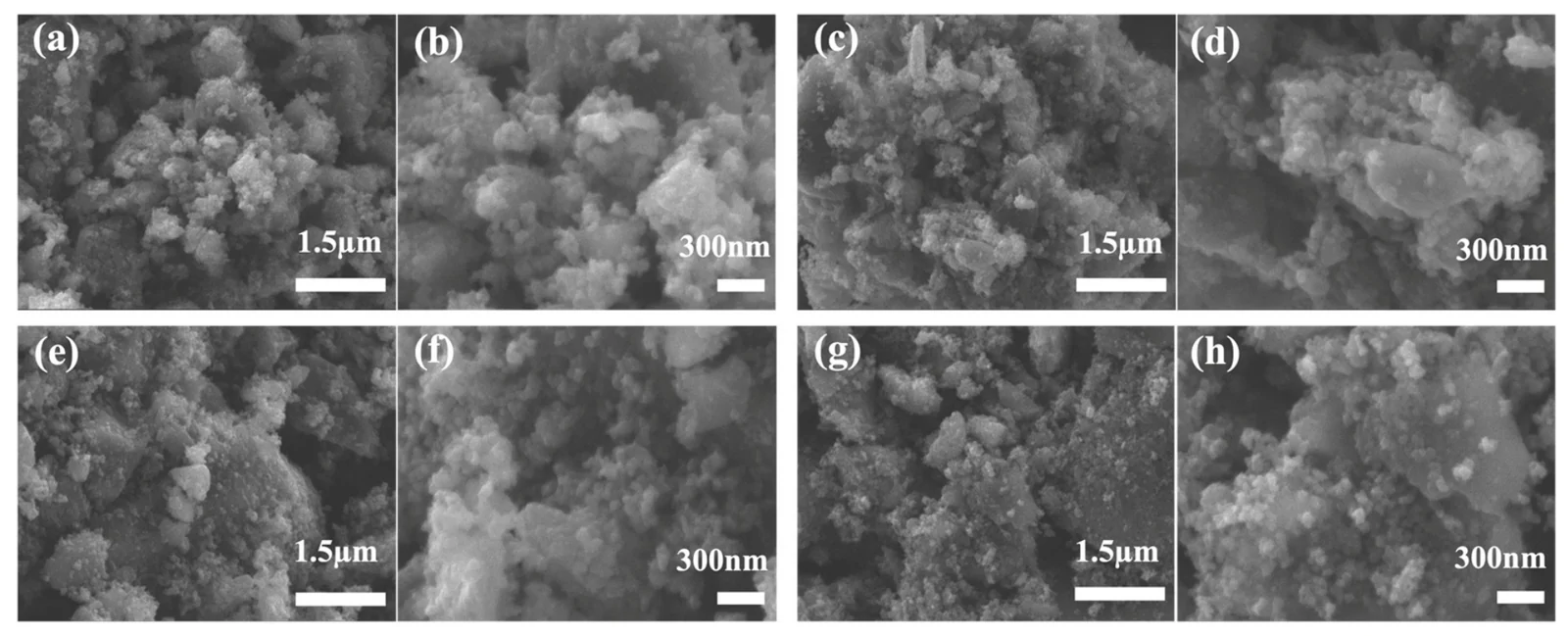
SEM micrographs of sulfonated PP-derived carbons carbonized at 700 °C (**a**,**b**), 800 °C (**c**,**d**), 900 °C (**e**,**f**) and 1000 °C (**g**,**h**), at low and high magnification (scale bars 1.5 µm and 300 nm, respectively).

As shown in Figure 3, all samples derived from waste PP share an irregular, granular morphology composed of micron-sized fragments, which is a characteristic appearance typical of carbons obtained from polymer precursors that have undergone chemical cross-linking via sulfonation [8,9].

The four samples appear broadly similar at these magnifications. PP-700 and PP-800 exhibit textured surfaces with loose aggregates of sub-micron particles adhering to larger angular fragments. This general appearance is retained at 900 and 1000 °C, where the fragments appear somewhat more compact and the adhering particulates rather less abundant, the surface particulates at 1000 °C beginning to merge into larger, plate-like structures consistent with the structural consolidation indicated by the XRD data [17,18].

Collectively, these observations indicate that increasing the carbonization temperature progressively consolidates the particle morphology, from loose sub-micron aggregates at 700–800 °C towards denser, plate-like domains at 1000 °C [19]. This trend is consistent with the decrease in surface area and pore volume measured by N_2_ physisorption, but the SEM data alone do not establish which sample offers the most favourable structure for lithium storage.

EDS elemental mapping was used to check whether the removal of sulfur indicated by XPS also holds over the particle interior, since XPS probes only the outermost few nanometers. The maps are shown in Figure S2. Carbon is distributed uniformly over the particles in every sample, and oxygen is also distributed uniformly, with some enrichment at particle edges. The map sum spectra give 94.9–96.3 wt% carbon and 3.7–5.1 wt% oxygen. Sulfur was below the detection limit in PP-700 and PP-900, and was quantified at 0.2 wt% in PP-800 and PP-1000, where the S Kα maps show only a uniform low-level signal with no sulfur-rich regions. Sulfur is therefore removed throughout the volume sampled by EDS and not only from the surface probed by XPS.

**Figure 4:**
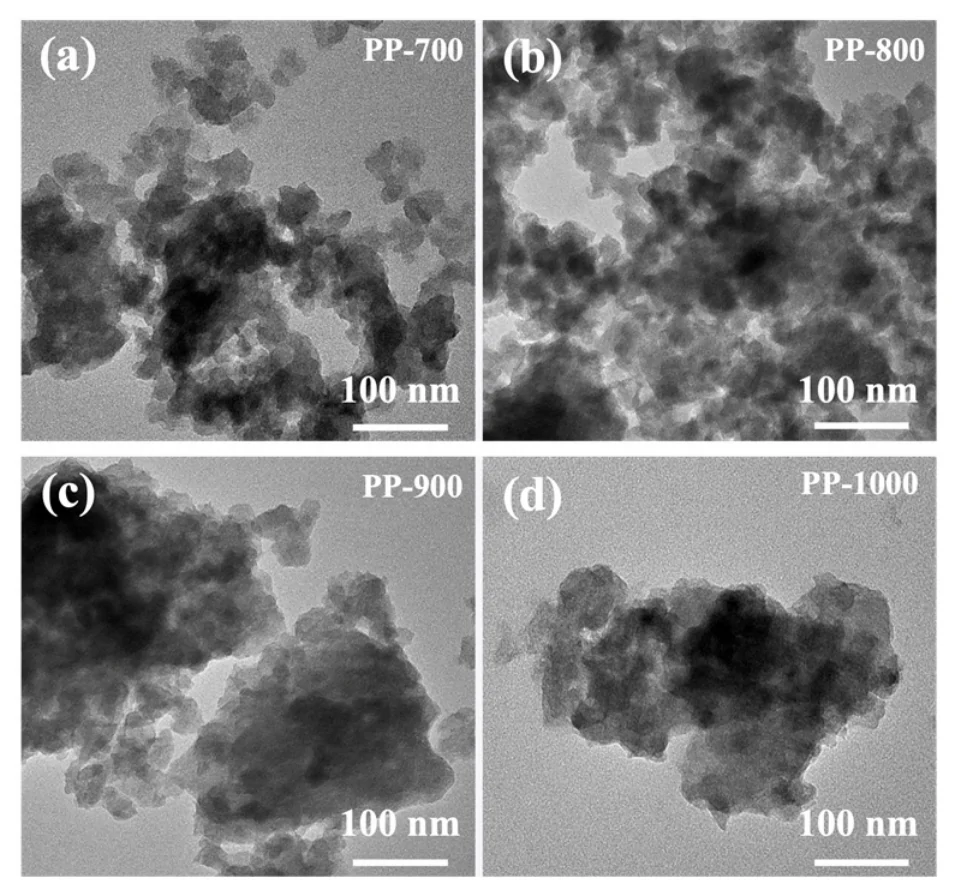
TEM images of (**a**) PP-700, (**b**) PP-800, (**c**) PP-900 and (**d**) PP-1000 (scale bar 100 nm).

TEM micrographs (Figure 4) reveal a similar overall morphology across the series, consisting of irregular, agglomerated carbon nanoparticle clusters at all four carbonization temperatures. Interparticle voids are visible in PP-700 and PP-800, while PP-900 and PP-1000 appear as denser, more compact agglomerates with less visible porosity. No lattice fringes could be reliably resolved in any of the samples. The TEM images corroborate the particle-scale morphology observed by SEM and the reduction in visible interparticle porosity at higher carbonization temperature, in agreement with prior reports on polypropylene-derived carbon anodes [8,10].

**Figure 5:**
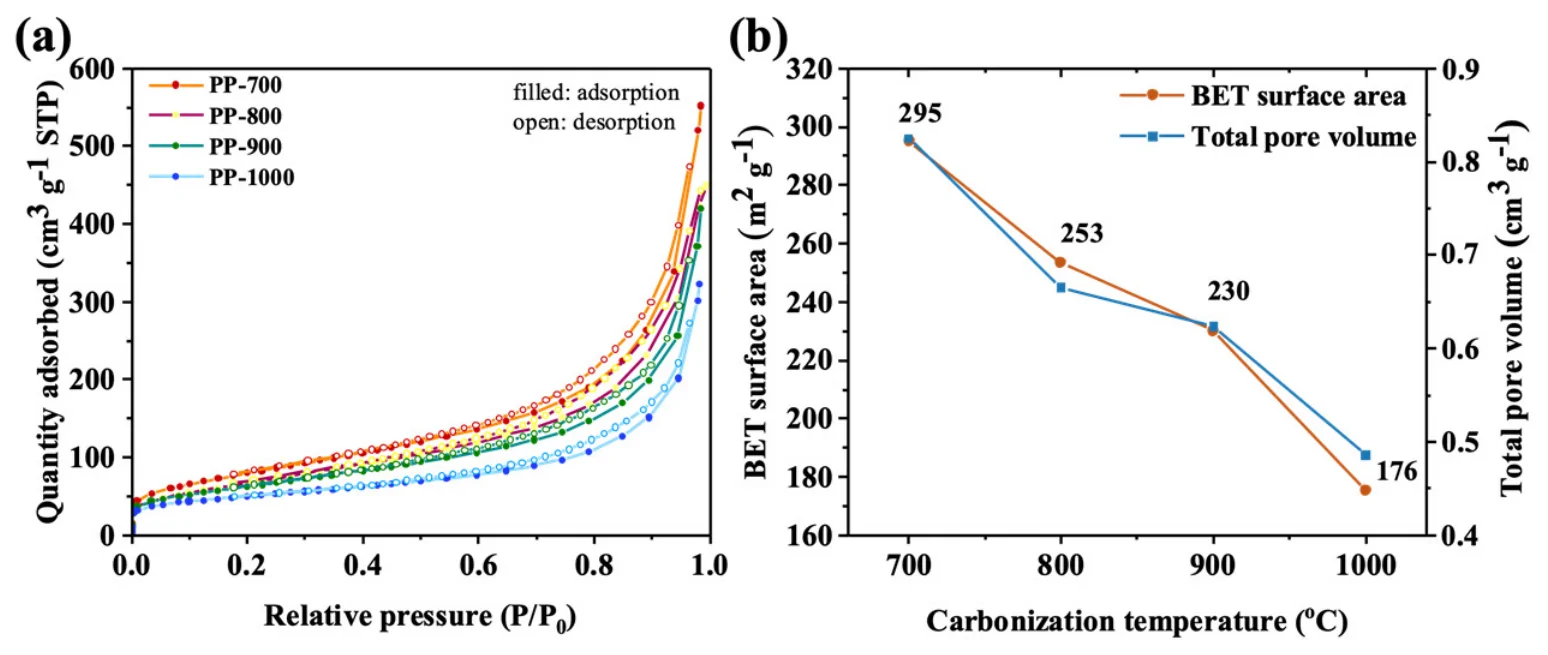
N_2_ physisorption analysis of the PP-derived carbons. (**a**) Adsorption–desorption isotherms at 77 K; (**b**) BET specific surface area and total pore volume as a function of carbonization temperature.

The porous texture of the PP-derived carbons was probed by N_2_ physisorption at 77 K (Figure 5a). All four samples display type IV(a) isotherms with a broad H3-type hysteresis loop, indicating a hierarchically micro-mesoporous texture. The steep uptake at low relative pressure (P/P0 < 0.05) corresponds to micropore filling, the gradual rise across the intermediate range to mesopores, and the sharp upturn as P/P0 → 1 to larger meso/macropores and interparticle voids [20]. Because these distributions are polymodal, a single median pore width does not describe them adequately, and the full distributions are therefore reported in Figure S1. Two-dimensional non-local density functional theory (2D-NLDFT, HS-2D-NLDFT carbon kernel) applied to the adsorption branch resolves a micropore population with modes near 0.5 and 0.9 nm, while BJH analysis of the same isotherms gives a broad mesopore population centred between 20 and 40 nm. The two ranges contribute very differently. Micropores, defined as pores narrower than 2 nm [20], account for only 5–8% of the total pore volume but 36–59% of the cumulative pore surface area, whereas mesopores carry 88–91% of the volume (Table 1). The texture is therefore hierarchical, with micropores contributing substantially to the surface area and mesopores dominating the pore volume.

The textural parameters are summarized in Table 1. The apparent BET specific surface area (Figure 5b) decreases monotonically with increasing carbonization temperature, from 295 m^2^ g^−1^ for PP-700 to 253, 230 and 176 m^2^ g^−1^ for PP-800, PP-900 and PP-1000, respectively, while the total pore volume falls from 0.825 to 0.486 cm3 g^−1^ over the same range. Whereas PP-700 and PP-800 consist of loose aggregates of sub-micron particles with abundant interparticle voids, PP-900 and PP-1000 progressively fuse into denser, plate-like domains with fewer resolvable voids. The accompanying shrinkage and partial collapse of the pore network at higher temperature (thermally induced densification) accounts for the systematic loss of surface area and pore volume [10].

**Table 1:** Textural properties of the PP-derived carbons from N_2_ physisorption.

Sample	SBET (m^2^ g^−1^)	V_total_ (cm^3^ g^−1^)	V_micro_ (cm^3^ g^−1^)	V_micro_/V_total_ (%)	S_micro_/S_total_ (%)
PP-700	295.3 ± 0.9	0.825	0.047	5.7	45
PP-800	253.4 ± 0.9	0.666	0.034	5.1	36
PP-900	230.3 ± 0.4	0.624	0.039	6.3	50
PP-1000	175.6 ± 1.0	0.486	0.039	8.1	59

SBET = BET specific surface area. V_total_, V_micro_ and the micropore fractions were obtained by 2D-NLDFT analysis of the adsorption branch. Micropores are pores narrower than 2 nm. Full distributions are given in Figure S1.

The structural evolution and degree of disorder in the PP-derived carbon samples were investigated using XRD and Raman spectroscopy, with the carbonization temperature found to significantly influence both the interlayer spacing and the microstructural organization.

**Figure 6:**
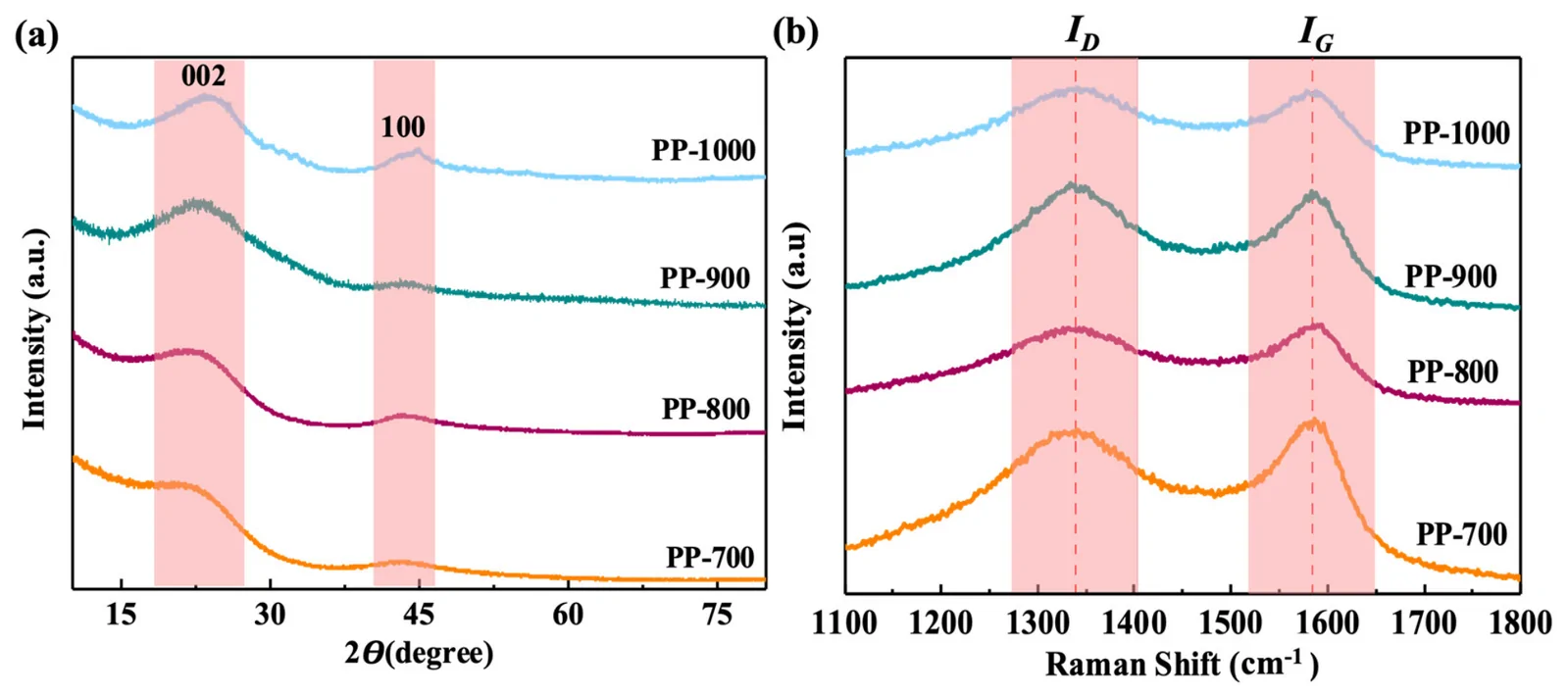
(**a**) X-ray diffraction patterns and (**b**) Raman spectra of carbonized samples (PP-700, PP-800, PP-900 and PP-1000).

The XRD patterns presented in Figure 6a exhibit two broad, low-intensity diffraction peaks centred at 2θ ≈ 22–24° and ≈44°, corresponding to the (002) and (100) planes of disordered carbon, respectively [21,22]. The (002) reflection shifts progressively to a higher angle with increasing carbonization temperature. The absence of sharp, high-intensity reflections confirms that the materials possess a low degree of long-range graphitic ordering and consist primarily of small, randomly oriented carbon layers [23].

The interlayer spacing (d_002_) was calculated from the (002) peak using Bragg’s Law, revealing a systematic structural contraction with increasing carbonization temperature (Table 2).

As carbonization temperature increases, d002 decreases systematically from 0.41 nm (PP-700) to 0.37 nm (PP-1000). All values considerably exceed that of ideal graphite (0.335 nm), confirming the presence of expanded, disordered carbon layers. This progressive contraction reflects thermal rearrangement and partial elimination of structural defects, resulting in more compact layer stacking [22]. This progressive interlayer contraction parallels the reduction in specific surface area and pore volume determined by N_2_ physisorption, both reflecting the densification of the carbon framework as the carbonization temperature increases. The smallest spacing in PP-1000 indicates the highest degree of structural ordering, whereas PP-700 retains the most expanded configuration, which would be expected to facilitate lithium intercalation [24,25].

These features place the present materials in the hard carbon (non-graphitizable) class rather than the soft carbon (graphitizable) class. Soft carbons pass through a fluid or plastic stage during pyrolysis, so their layers are able to align and grow into extended, well-stacked graphitic domains on further heat treatment, and their interlayer spacing approaches the 0.335 nm of graphite. The sulfonated PP precursor used here is instead cross-linked and infusible before carbonization begins, and the resulting carbons retain d002 values between 0.41 and 0.37 nm together with a broad, weak (002) reflection up to 1000 °C, which indicates small, turbostratically stacked domains with no long-range order. The same behaviour has been reported for other PP-derived carbons, which do not graphitize even after heat treatment at 2400 °C [9]. The materials are therefore described as hard carbons throughout, and the contraction of d002 with increasing temperature is interpreted as local ordering within a persistently non-graphitizable framework, not as the onset of graphitization.

The microstructural disorder and defect levels were further quantified using Raman spectroscopy (Figure 6b). All samples display two prominent characteristic bands: the D-band (at ~1350 cm^−1^), associated with structural defects, edge sites, and sp^3^-hybridized carbon atoms, and the G band (at ~1580 cm^−1^), representing the in-plane vibration of sp^2^-hybridized carbon within graphitic domains [14,26,27]. The intensity ratio I_D_/I_G_, evaluated here from the peak heights of the fitted D and G bands, is widely used as an empirical indicator of the degree of structural disorder, and the values obtained are summarized in Table 2. In highly disordered carbons, this ratio can increase either with defect density or with the size of the sp^2^ clusters, so it is used here as a comparative indicator between samples rather than as a quantitative defect density [27].

The I_D_/I_G_ ratio varies non-monotonically with carbonization temperature and passes through a maximum at 900 °C. PP-900 gives the highest value of the series (1.12), PP-700 a lower value (0.94) despite its wider interlayer spacing, PP-800 the lowest (0.88) and PP-1000 an intermediate value (0.92). One interpretation, consistent with trends reported for other hard carbons, is that the removal of heteroatoms and residual sp^3^ carbon between 700 and 800 °C reduces the intensity of the D band, that the growth and rearrangement of small sp^2^ clusters increases it at 900 °C, and that cluster coalescence and defect annealing reduce it again at 1000 °C [24]. It is worth noting that the maximum in I_D_/I_G_ for PP-900 does not coincide with the highest surface area, which is found for PP-700 (Table 1), so the specific surface area is not the structural parameter that tracks the electrochemical capacity reported in Section 3.2. A high surface area, as in PP-700, would in addition be expected to promote more extensive SEI formation and to lower the initial Coulombic efficiency (CE) [13].

The surface composition and chemical state of the carbons were examined by XPS. Only carbon, oxygen and sulfur are detected in the survey spectra, and the corresponding atomic concentrations are given in Table 3. Carbon accounts for 92–94 at% in every sample. The oxygen content lies between 6.3 and 7.5 at% and increases slightly with carbonization temperature, which is most simply explained by re-oxidation of the carbon surface on exposure to air after carbonization rather than by retention of the original sulfonate groups. Sulfur, in contrast, is present only at trace level: 0.15–0.20 at% in PP-700, PP-800 and PP-1000, and below the detection limit in PP-900. The sulfonating agent is therefore almost completely removed from the carbon surface during carbonization, which is the point left open by the TGA-MS data discussed above [28].

**Table 3:** Surface elemental composition of the PP-derived carbons determined from XPS survey spectra (n.d. = not detected).

Sample	C (at%)	O (at%)	S (at%)
PP-700	93.6	6.3	0.18
PP-800	92.9	7.0	0.15
PP-900	92.8	7.3	n.d.
PP-1000	92.3	7.5	0.20

**Figure 7:**
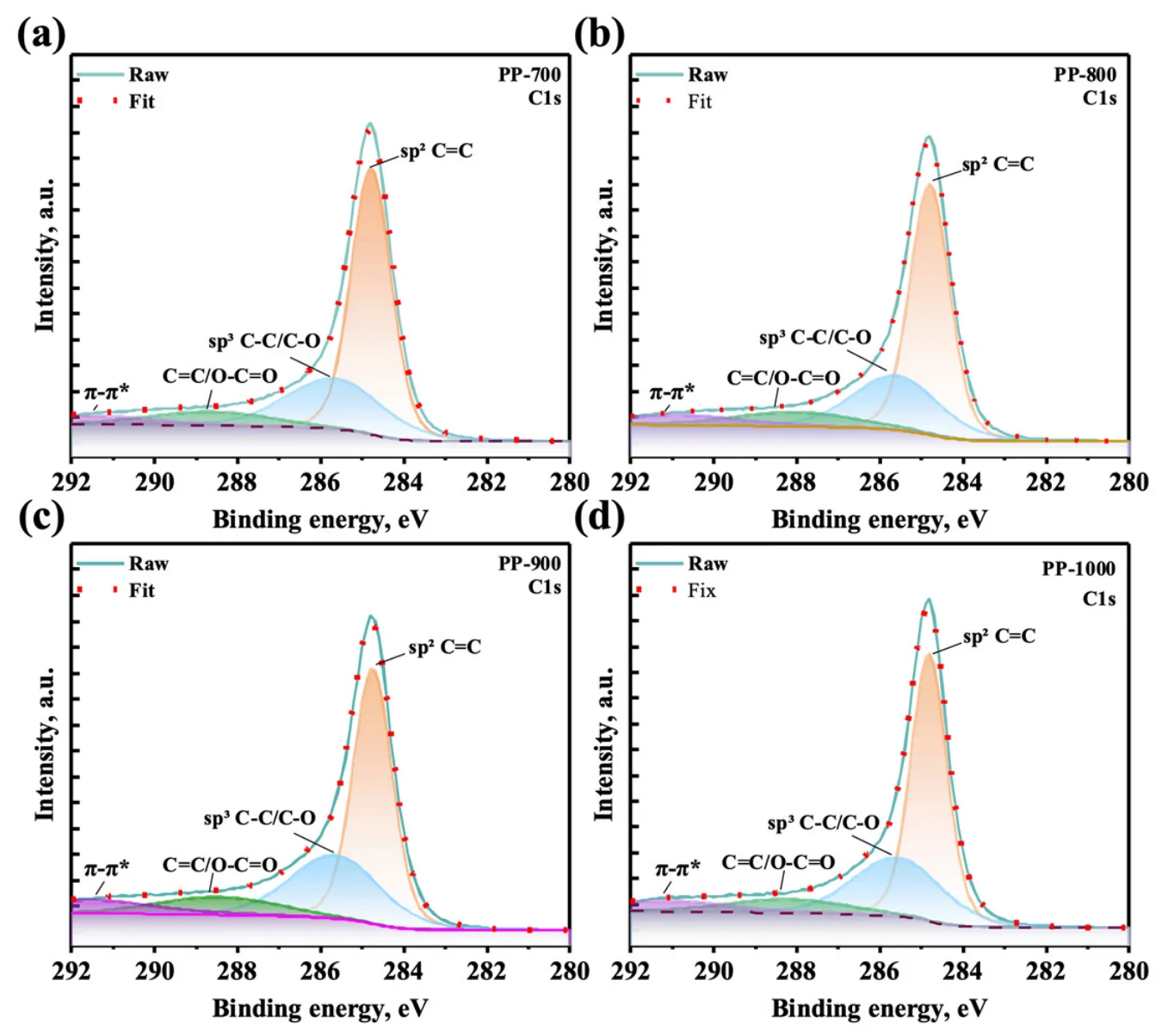
High-resolution C 1s XPS spectra of (**a**) PP-700, (**b**) PP-800, (**c**) PP-900 and (**d**) PP-1000, showing the measured data, the fitted components and the resulting envelope.

The high-resolution C 1s spectra (Figure 7) were fitted with four components: a main line at 284.8 eV assigned to sp^2^ carbon, a component at 285.6 eV assigned to sp^3^ carbon and to carbon singly bonded to oxygen (C-O), a component at 288.0–288.6 eV assigned to carbonyl and carboxyl carbon (C=O and O-C=O), and a broad feature at 290.8–291.5 eV assigned to the π-π* shake-up satellite characteristic of aromatic carbon [8,9]. The relative area of the shake-up satellite increases from 6.8% of the total C 1s intensity in PP-700 to 8.2% in PP-800 and 9.9% in PP-900, and decreases slightly to 9.0% in PP-1000. This ordering follows the same non-monotonic trend as the ID/IG ratio, with a maximum at 900 °C, and is consistent with the growth of aromatic clusters up to that temperature. The remaining components vary little across the series (sp^2^ carbon 51–58%, combined sp^3^ C and C-O 26–29%, and C=O together with O-C=O 9–11% of the total C 1s intensity), so the shake-up satellite provides the clearest compositional distinction between the samples as seen in Table 4.

**Table 4:** C 1s components of the PP-derived carbons, as a percentage of the total fitted C 1s peak area. The O/C ratio is from the survey spectra in Table 3.

Component	Binding Energy (eV)	PP-700	PP-800	PP-900	PP-1000
sp^2^ C=C	284.8 ± 0.1	57.8	55.5	50.7	53.1
sp^3^ C and C-O	285.6 ± 0.1	26.5	26.1	28.7	28.5
C=O and O-C=O	288.0–288.6	8.9	10.2	10.7	9.5
π-π* shake-up	290.8–291.5	6.8	8.2	9.9	9.0
sp^2^/(sp^3^ + C–O) ratio	-	2.18	2.13	1.77	1.86
O/C atomic ratio (survey)	-	0.067	0.075	0.078	0.081

Taken together, PP-900 combines an intermediate interlayer spacing (0.38 nm), an intermediate surface area, the highest ID/IG ratio of the series and the most intense π-π* shake-up satellite in the C 1s spectrum, whereas PP-1000 shows the most contracted interlayer spacing, the lowest surface area and a reduced ID/IG ratio, consistent with thermally induced compaction and the development of low-defect graphitic domains [11,22,24,28,29,30]. The structural trends are consistent with the electrochemical results presented in Section 3.2, in which PP-900 delivers the highest capacity at low current density and PP-1000 a lower capacity than PP-900 despite its greater structural order.

### 3.2. Electrochemical Performance

**Figure 8:**
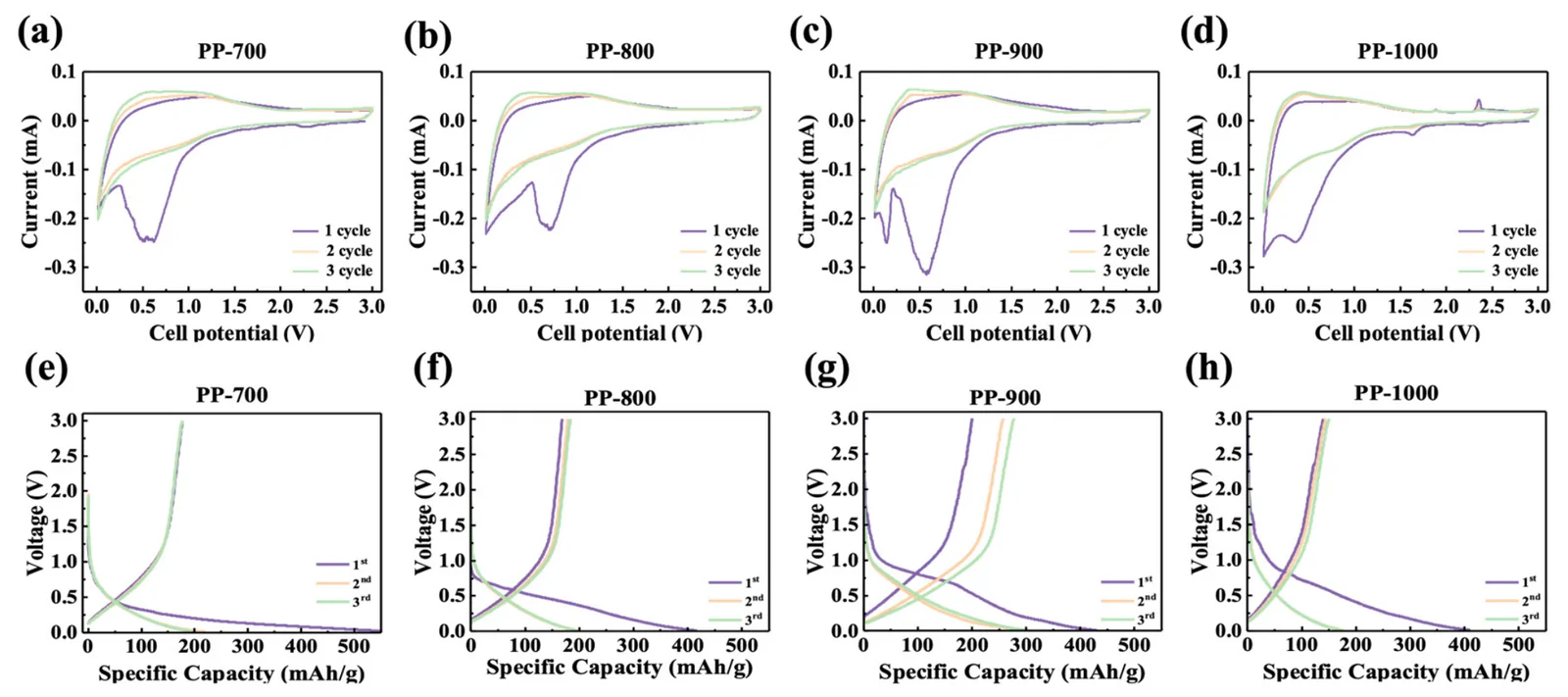
Electrochemical performance of PP-derived carbon electrodes: (**a**–**d**) cyclic voltammetry curves recorded at a scan rate of 0.1 mV s^−1^ and (**e**–**h**) galvanostatic charge–discharge profiles measured at 0.1 A g^−1^ for PP-700, PP-800, PP-900 and PP-1000, respectively.

The electrochemical performance of the PP-derived carbon materials was investigated using cyclic voltammetry (CV) and galvanostatic charge–discharge measurements. These techniques provide insights into lithium-ion storage mechanisms and the influence of carbonization temperature on the electrochemical behaviour of the prepared electrodes. The corresponding electrochemical results are presented in Figure 8.

The cyclic voltammetry profiles of the carbon electrodes (PP-700 to PP-1000) were recorded at a scan rate of 0.1 mV s^−1^ over a potential window of 0.01–3.0 V (vs. Li^+^/Li). The corresponding CV curves are presented in Figure 8a–d. During the initial cathodic sweep, a broad reduction peak appearing at approximately 0.7–0.8 V is observed, which is attributed to electrolyte decomposition and the formation of a solid electrolyte interphase (SEI) layer on the carbon surface [31]. The disappearance of this feature in subsequent cycles indicates the establishment of a stable SEI film after the first cycle. At lower potentials (below ~0.2 V), a pronounced increase in cathodic current is observed, corresponding to lithium-ion insertion into the disordered carbon matrix, including interlayer sites associated with the expanded graphitic domains identified by XRD [24,32]. In the anodic sweep, a corresponding oxidation peak below 0.5 V is attributed to the reversible deintercalation of lithium ions from the carbon structure. The strong overlap between the second and third cycles for all samples indicates that the process becomes largely reversible once the SEI has formed [31].

The charge–discharge profiles were recorded at a current density of 0.1 A g^−1^. The corresponding curves are shown in Figure 8e–h. All samples exhibit a typical two-stage lithium storage behaviour characteristic of disordered carbon materials, consisting of a sloping region and a low-potential plateau. The sloping region (1.5–0.1 V) is associated with lithium-ion adsorption on surface defects, edges and vacancy sites within the carbon framework [24,32]. The relative contribution of this region is generally taken to depend on the density of defects and on the degree of structural disorder [13]. The plateau region below 0.1 V corresponds to lithium-ion intercalation into nanopores and between graphitic-like domains formed during carbonization. The extent of this plateau is influenced by the interlayer spacing determined from XRD [33].

The initial CE is low for all samples, primarily because of irreversible lithium consumption during SEI formation and trapping of lithium ions at defect-rich sites. The specific discharge capacity shows a clear dependence on carbonization temperature. As the temperature increases from 700 °C to 900 °C, the capacity increases, and it decreases again at 1000 °C. A plausible explanation is that an intermediate carbonization temperature provides the most favourable combination of accessible storage sites and interlayer spacing, whereas the structural ordering and interlayer contraction that occur at 1000 °C reduce the number of sites available for lithium [13,32]. This reflects the balance between structural disorder, which increases the number of lithium storage sites, and graphitization, which improves electronic conductivity but reduces their availability [13,24].

**Figure 9:**
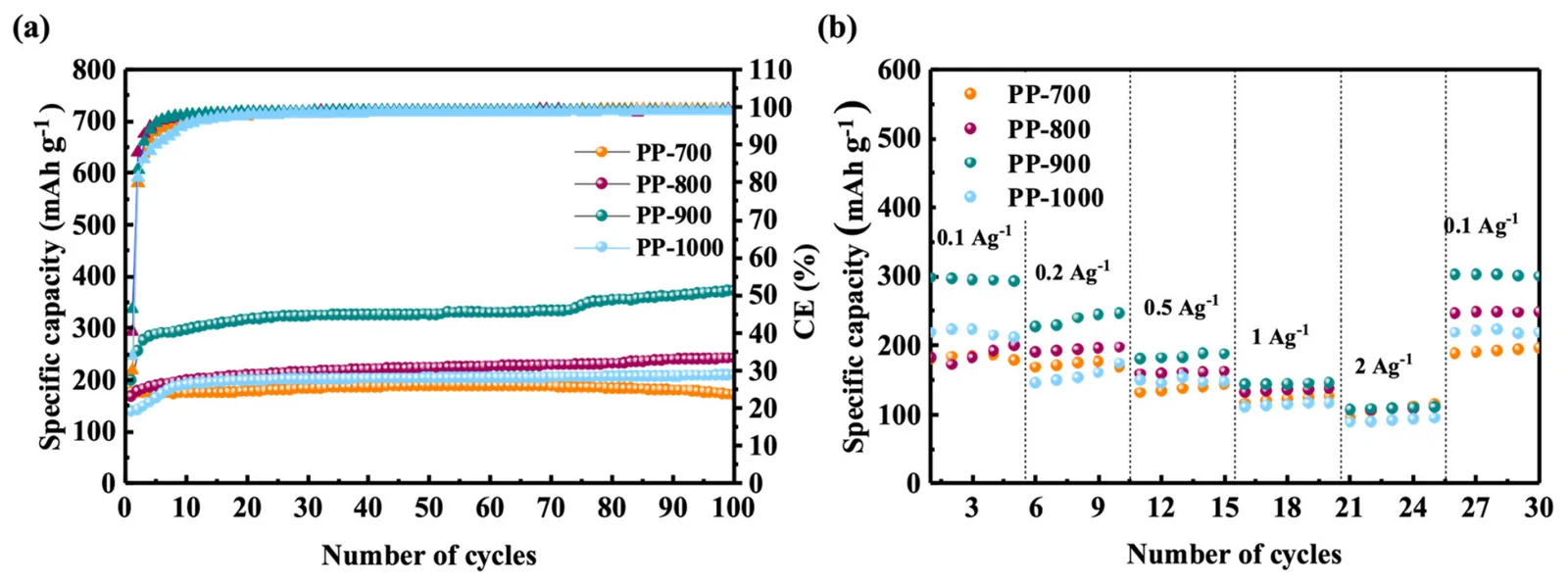
Electrochemical cycling behaviour of PP-700, PP-800, PP-900 and PP-1000 electrodes: (**a**) cycling performance and Coulombic efficiency at 0.1 A g^−1^ over 100 cycles; (**b**) rate capability at current densities from 0.1 to 2 A g^−1^.

The long-term cycling stability and rate capability of the PP-derived carbon materials were evaluated to assess their suitability as LIB anodes. The corresponding cycling performance and rate capability results are presented in Figure 9a,b.

The cycling performance of the electrodes was investigated at a current density of 0.1 A g^−1^ over 100 charge–discharge cycles. The electrodes exhibit markedly different cycling evolution. PP-800, PP-900 and PP-1000 undergo progressive capacity activation during cycling, whereas PP-700 shows an initial increase followed by moderate fading (Figure 9a). The relatively low initial CE is attributed to irreversible lithium consumption associated with SEI formation and lithium trapping at defect-rich sites within the carbon structure [13]. Following the initial stabilization period, the CE remains high and nearly constant.

Among the investigated samples, the carbon obtained at 900 °C exhibits the highest reversible capacity throughout the cycling test, increasing from 200.6 mAh g^−1^ at the 1st cycle to 373.1 mAh g^−1^ at the 100th cycle, a gain of 86%. This behaviour is not stable cycling in the usual sense. The capacity of PP-900 does not reach a plateau within 100 cycles but continues to rise, which is characteristic of progressive electrochemical activation [13,24]. The remaining samples deliver lower capacities but show a qualitatively similar evolution. PP-800 rises from 168.4 to 242.6 mAh g^−1^ (+44%) and PP-1000 from 139.8 to 209.7 mAh g^−1^ (+50%), which is a similar though somewhat less pronounced activation, whereas PP-700 reaches a maximum of 190.2 mAh g^−1^ at cycle 57 before fading to 172.2 mAh g^−1^ by cycle 100. In addition, the first-cycle Coulombic efficiency ranges from 30.2% to 46.7% across the series, exceeds 95% within the first ten cycles for every sample, and stabilizes at 99.1–99.7% by the 100th cycle. The overall cycling behaviour demonstrates that the electrochemical properties are strongly influenced by the carbonization temperature, which governs the development of defects, porosity, and interlayer structure.

A plausible origin of this capacity increase is the progressive activation of the micropore network. Micropores narrower than 2 nm carry 36–59% of the cumulative pore surface area (Table 1, Figure S1), and a carbonate-based electrolyte wets pores of this width only slowly, so continued electrolyte penetration during cycling would gradually increase the electrochemically accessible surface and hence the measured capacity [13,24]. Other processes may contribute over the same period, including continued evolution of the SEI [34], gradual reduction in residual oxygen-containing surface groups [28], and slow rearrangement of the disordered carbon framework during repeated lithiation [13,24].

Values of 30.2–46.7% are low compared with typical hard carbons, for which 50–80% is commonly reported [13,24], and represent the most serious limitation of the present materials. The high apparent BET surface areas of 176–295 m^2^ g^−1^ provide a correspondingly large area over which the electrolyte is reduced during SEI formation, and the large fraction of that area residing in micropores (36–59%, Table 1) accentuates this effect, since electrolyte that penetrates narrow pores decomposes within them and may block the pore mouths irreversibly [24,28]. The residual oxygen-containing surface groups, which account for 6.3–7.5 at% of the surface composition (Table 3), can react irreversibly with lithium in the first cycle, whereas the residual sulfur content is too low (≤0.2 at%) to contribute appreciably [28]. Established mitigation strategies include reducing the accessible surface area by carbonization at higher temperature or by carbon coating, the use of film-forming electrolyte additives such as fluoroethylene carbonate, and pre-lithiation of the anode [13,24].

Table 5 places these results alongside carbons obtained from comparable plastic waste precursors. The reversible capacity of PP-900 is competitive within this group and exceeds that reported for carbons from recycled PP separators and from waste plastic bags, while remaining below the maximum discharge capacity reported for sulfonated PP mask waste in a lithium cell [8]. The initial Coulombic efficiency of the present materials is at the low end of the group. It is close to the value reported for a plastic-bag-derived carbon in a lithium cell [35] and to the 48.4% reported for the sulfonation-only route in [8], but clearly below the values of 68–73% reported for PP-derived hard carbons in sodium cells [9,10], where the different electrolyte chemistry and the smaller contribution of surface-film formation both favour a higher initial efficiency. Within the lithium-cell comparison, the ordering follows the accessible surface area, which supports the interpretation given above.

The rate capability of the electrodes was evaluated by progressively increasing the current density from 0.1 to 2 A g^−1^ (Figure 9b, Table 6). For all samples, the specific capacity decreases with increasing current density. PP-900, for example, falls from 293.6 mAh g^−1^ at 0.1 A g^−1^ to 111.1 mAh g^−1^ at 2 A g^−1^, reflecting kinetic limitations associated with lithium-ion diffusion and charge transfer within the carbon framework [32]. PP-900 delivers the highest capacity from 0.1 to 1 A g^−1^, but at 2 A g^−1^ PP-700 (115.3 mAh g^−1^), PP-900 (111.1 mAh g^−1^) and PP-800 (110.4 mAh g^−1^) are equivalent to within a few per cent. Capacity retention going from 0.1 to 2 A g^−1^ follows the opposite order: 65% for PP-700, 55% for PP-800, 45% for PP-1000 and 38% for PP-900, so the advantage of PP-900 lies at low and moderate current densities. When the current density is returned to 0.1 A g^−1^, all electrodes recover their initial capacity, indicating that high-rate operation does not cause irreversible degradation of the carbon framework [36]. The recovered values in fact exceed the initial ones, by 10% for PP-700, 24% for PP-800, 2% for PP-900 and 1% for PP-1000, showing that the activation observed in the cycling test (Figure 9a) continues during the rate test.

The difference in rate behaviour is qualitatively consistent with the textural and structural data. PP-700 combines the largest apparent surface area (295 m^2^ g^−1^), the largest pore volume (0.825 cm^3^ g^−1^) and the widest interlayer spacing (0.41 nm) of the series, all of which shorten the solid-state diffusion path and favour storage at readily accessible surface and near-surface sites. Capacity of this kind is intrinsically less rate-limited than storage in nanopores and between graphitic-like domains. On this reading, the larger low-rate capacity of PP-900 would be associated with storage sites that are less accessible on short timescales.

**Figure 10:**
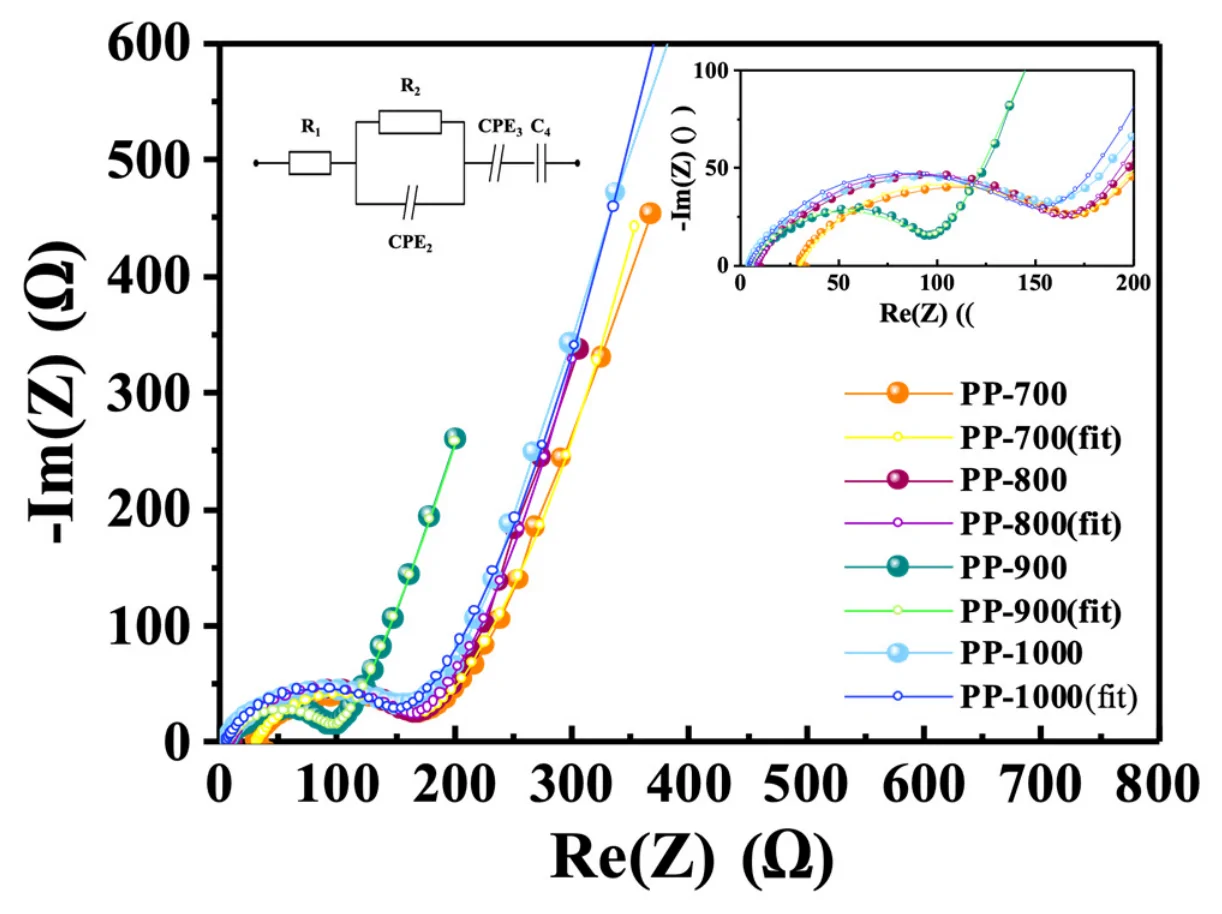
Nyquist plots of the PP-700, PP-800, PP-900 and PP-1000 electrodes recorded after cycling, with the fitted spectra overlaid, and the equivalent circuit used for the fitting.

To examine the electrode kinetics of the carbonized samples, electrochemical impedance spectroscopy (EIS) was performed after the cyclic voltammetry cycles described above, so that an SEI layer had already formed on each electrode. The Nyquist plots of PP-700, PP-800, PP-900 and PP-1000 are shown in Figure 10. All four spectra have the same overall shape. At high frequency, the curve begins on the real axis at a small positive value, which corresponds to the series resistance of the cell. This is followed by a single flattened semicircle in the high- and middle-frequency range, and then by an inclined line at low frequency that becomes steeper towards the end of the measurement, reaching an angle of about 70° to the real axis in every sample. Only one semicircle is resolved in each spectrum, so the surface-film and charge-transfer contributions are not separated here and are described together by a single arc [33,34].

The spectra were fitted with the equivalent circuit R_1_ + (R_2_/CPE_2_) + CPE_3_ + C_4_, shown in Figure 10. R_1_ is the series resistance of the cell, which includes the electrolyte, the separator, the current collector and the electronic resistance of the carbon electrode, and it determines the point at which the spectrum meets the real axis at high frequency. R_2_ is the interfacial resistance and corresponds to the diameter of the semicircle; describing how easily lithium ions cross the boundary between the electrode and the electrolyte: the larger the semicircle, the more difficult this step. It is placed in parallel with a constant phase element, CPE_2_, which represents the charge stored at the electrode surface. A constant phase element is used instead of an ideal capacitor because the carbon surface is porous and chemically non-uniform, and this is what makes the semicircles flattened rather than perfect half-circles [37]. CPE_3_ and C_4_ are connected in series and together describe the low-frequency part of the spectrum, CPE_3_ accounting for the inclined line and C_4_ for its steepening at the lowest frequencies. All four spectra were fitted over the same frequency range and with the same weighting (|Z|^2^). The fitted parameters and the goodness-of-fit values are given in Table 7. The weighted goodness of fit χ^2^/|Z|^2^ lies between 0.112 and 0.173 and the corresponding χ/√N between 1.31 and 6.10 Ω, and the fitted spectra are overlaid on the measured data in Figure 10.

The series resistance R_1_ decreases as the carbonization temperature increases, from 29.8 Ω for PP-700 to 7.7 Ω for PP-800, 4.6 Ω for PP-900 and 4.0 Ω for PP-1000. This is seen directly in Figure 10 as a shift in the starting point of the spectrum towards the origin. Since all the cells were assembled in the same way and with the same electrolyte, separator and electrode size, this difference is attributed mainly to the electronic resistance of the carbon, which decreases as carbonization proceeds. It is consistent with the XRD results, where the interlayer spacing d002 decreases from 0.41 nm for PP-700 to 0.37 nm for PP-1000 (Table 2), indicating more ordered stacking of the carbon layers. PP-700 is clearly separated from the other three samples in this respect, whereas PP-900 and PP-1000 differ only slightly.

The semicircles differ noticeably in size. PP-900 gives the smallest arc, which closes on the real axis at about 96 Ω, and the lowest interfacial resistance of the series, R_2_ = 92 Ω. The other samples give larger and rather similar values, R_2_ = 132 Ω for PP-700, 143 Ω for PP-1000 and 153 Ω for PP-800, with their arcs closing between about 154 and 170 Ω. Lithium ions therefore appear to cross the electrode–electrolyte interface most easily in PP-900, while the other three samples behave similarly to one another. The exponent of CPE_2_ is almost the same for all four electrodes (0.68–0.70), showing that the degree of flattening of the arc is comparable in every case, as expected for the geometrically and chemically heterogeneous surface indicated by N_2_ physisorption (Table 1, Figure S1). The magnitude of CPE_2_ is largest for PP-900 (59.7 μF s^a−1^) and smallest for PP-1000 (24.9 μF s^a−1^), with PP-700 and PP-800 at 28.3 and 32.8 μF s a^−1^. This ordering does not follow the BET surface areas, which decrease steadily from PP-700 to PP-1000 (Table 1).

At low frequency, all four samples show the same qualitative behaviour, an inclined line that steepens towards the end of the measurement, and the fitted exponent of CPE_3_ lies between 0.47 and 0.61. The capacitance C4 is the least well-defined parameter of the model in every sample, because the fitted frequency range ends before this part of the response is fully developed, and its values are therefore not discussed further. Overall, the impedance data show that the carbonization temperature controls the series resistance of the electrodes, and that PP-900 combines a low series resistance with the smallest interfacial resistance of the series. This is consistent with PP-900 delivering the highest capacity at low and moderate current densities (Table 6). The measurements were made at a single state after cycling, however, and do not by themselves account for the rate capability ranking discussed above.

Overall, the electrochemical results indicate that carbonization temperature strongly influences lithium storage performance, but that no single sample is optimal in every respect. PP-900 provides the highest capacity at low current density and the largest capacity gain during cycling, whereas PP-700 shows the best capacity retention at high current density and PP-800 the largest recovery on returning to a low rate. The observed behaviour correlates qualitatively with the structural characteristics identified by XRD, Raman spectroscopy and N_2_ physisorption. The impedance results discussed above show that the series resistance is governed by the carbonization temperature and that PP-900 has the smallest interfacial resistance of the series.

## 4. Conclusions

In this work, waste PP was successfully converted into hard carbon (non-graphitizable) anode materials for LIBs through sulfuric acid-assisted stabilization followed by carbonization at temperatures ranging from 700 to 1000 °C. The sulfonation treatment effectively transformed the thermally unstable PP into a cross-linked precursor capable of yielding carbon during high-temperature processing. Structural characterization demonstrated that carbonization temperature significantly influenced the microstructure of the resulting carbons. XRD analysis revealed a progressive decrease in interlayer spacing with increasing carbonization temperature, although d002 remained between 0.41 and 0.37 nm in every sample, confirming that the carbons stay non-graphitizable across the whole temperature range, while Raman spectroscopy gave the highest I_D_/I_G_ ratio for the sample carbonized at 900 °C. XPS showed that the carbons retain 6.3–7.5 at% oxygen at the surface but almost no sulfur (≤0.2 at%), indicating that sulfur is essentially absent from the final carbons; the data establish this endpoint but do not identify the stage at which, or the chemical form in which, the sulfur is removed.

Electrochemical investigations confirmed that the carbonization temperature strongly affected lithium storage performance. Among the investigated samples, the material prepared at 900 °C exhibited the highest reversible capacity at 0.1 A g^−1^, reaching 373.1 mAh g^−1^ at the 100th cycle. Its capacity increased progressively throughout the test rather than remaining constant, and this behaviour is better described as electrochemical activation than as stable cycling. The rate performance followed a different ordering: PP-900 showed the lowest capacity retention of the series at 2 A g^−1^ (38%, against 65% for PP-700), so that its advantage is confined to low and moderate current densities. All samples showed low first-cycle Coulombic efficiencies of 30.2–46.7%, which is the principal limitation of the materials in their present form. After the initial cycles, the Coulombic efficiency stabilized at 99.1–99.7%, indicating largely reversible charge–discharge behaviour thereafter.

These findings show that waste PP can serve as a precursor for carbon anode materials, and highlight the influence of carbonization temperature on electrochemical performance. Impedance spectroscopy showed that the series resistance of the electrodes falls steadily as the carbonization temperature increases, and that PP-900 has the smallest interfacial resistance of the series, which is consistent with its higher capacity at low and moderate current densities. Reducing the first-cycle irreversible loss remains the most important target for improving these materials.

## Figures and Tables

**Table 2:** Summary of XRD-derived interlayer spacing and Raman ID/IG ratios for carbonized PP samples.

Samples	D_002_ (nm)	I_D_/I_G_
PP-700	0.41	0.94
PP-800	0.39	0.88
PP-900	0.38	1.12
PP-1000	0.37	0.92

**Table 5:** Electrochemical characteristics of the present materials compared with carbons from other plastic waste. Values are as reported in the cited works. Lithium and sodium cells are not directly comparable.

Precursor	Stabilization	Ion	Reversible Capacity	Rate Capability	Initial CE (%)	Ref.
PP disposable masks	Sulfonation	Li	438.1 mAh g^−1^ (max. discharge)	56.5% retained at 5C	48.4	[8]
PP disposable masks	Sulfonation	Na	340 mAh g^−1^ at 0.01 A g^−1^	53% retained at 1 A g^−1^	68.2	[9]
Recycled PP separators	Sulfonation	Na	293.0 mAh g^−1^ at 0.2C	77.1 mAh g^−1^ at 10C	72.6	[10]
Waste plastic bags	Autogenic reaction	Li	240 mAh g^−1^ at 1C	not reported	40 (60% first-cycle loss)	[35]
Waste PP packaging (this work)	Sulfonation	Li	373.1 mAh g^−1^ at 0.1 A g^−1^ (100th cycle)	111.1 mAh g^−1^ at 2 A g^−1^	30.2–46.7	this work

**Table 6:** Rate capability of the PP-derived carbon electrodes.

Sample	0.1 A g^−1^	0.2 A g^−1^	0.5 A g^−1^	1 A g^−1^	2 A g^−1^	Recovered at 0.1 A g^−1^
PP-700	178.6	169.6	143.7	127.3	115.3	196.8
PP-800	200.8	197.3	162.8	137.7	110.4	249.2
PP-900	293.6	247.3	188.1	146.8	111.1	300.4
PP-1000	212.6	174.5	148.2	117.7	94.7	214.6

Capacities are given in mAh g^−1^. Capacity retention on going from 0.1 to 2 A g^−1^: PP-700 64.6%, PP-800 55.0%, PP-900 37.8%, PP-1000 44.5%.

**Table 7:** Equivalent circuit parameters fitted to the impedance spectra in Figure 10. Q and a are the magnitude and exponent of the corresponding constant phase element.

Parameter	PP-700	PP-800	PP-900	PP-1000
R_1_ (Ω)	29.78	7.74	4.62	4.02
R_2_ (Ω)	131.98	153.11	92.48	143.28
Q_2_ (μF s^a^_^2^_^−1^)	28.34	32.76	59.71	24.94
a_2_	0.677	0.676	0.676	0.705
Q_3_ (μF s^a^_^3^_^−1^)	4.80	6.31	7.49	3.84
a_3_	0.473	0.511	0.607	0.512
C_4_ (μF)	5.97	8.63	13.87	4.10
χ^2^/|Z|^2^	0.126	0.112	0.126	0.173
χ/√N (Ω)	3.98	2.59	1.31	6.10

## Data Availability

The original contributions presented in this study are included in the article/Supplementary Material. Further inquiries can be directed to the corresponding authors.

## Supplementary Materials

The following supporting information can be downloaded at: https://www.mdpi.com/article/10.3390/ma19194153/s1, Table S1: Masses recorded for the twelve individual sulfonation and carbonization experiments. Table S2: Sulfonation mass gain, carbonization yield and overall yield for each carbonization temperature, as mean and standard deviation over the three independent replicate syntheses in Table S1. Figure S1: Pore size distributions of the PP-derived carbons from the nitrogen isotherms measured at 77 K. Figure S2: EDS elemental maps of the PP-derived carbons. Columns, from left to right: electron image, C Kα, O Kα and S Kα.

## Abbreviations

The following abbreviations are used in this manuscript:

PP	Polypropylene
BET	Brunauer–Emmett–Teller
BJH	Barrett–Joyner–Halenda
CE	Coulombic efficiency
CPE	Constant phase element
CV	Cyclic voltammetry
DEC	Diethyl carbonate
DMC	Dimethyl carbonate
EC	Ethylene carbonate
EDS	Energy-dispersive X-ray spectroscopy
EIS	Electrochemical impedance spectroscopy
FTIR	Fourier-transform infrared spectroscopy
LIB	Lithium-ion battery
MS	Mass spectrometry
NLDFT	Non-local density functional theory
NMP	N-methyl-2-pyrrolidone
PVDF	Polyvinylidene fluoride
SEI	Solid electrolyte interphase
SEM	Scanning electron microscopy
TEM	Transmission electron microscopy
TGA	Thermogravimetric analysis
XPS	X-ray photoelectron spectroscopy
XRD	X-ray diffraction
