# Supplementary material for: Upcycling Polypropylene Waste into Carbon Anode Materials for Lithium-Ion Batteries
Source: Materials (Basel). 2026 Sep 29;19(19):4153. doi: 10.3390/ma19194153 (PMC13666341; doi:10.3390/ma19194153)
Supplement: Supplementary file 1 [file materials-19-04153-s001.zip › materials-4560805-supplementary.pdf]

Supplementary Materials

Upcycling polypropylene waste into carbon anode materials for lithium-ion batteries

Yryszhan Tashenova, Kuanysh Kurtibay, Dossym Yeskozha, Aliya Mukanova, Sung-Soo Kim, Arailym Nurpeissova

Table S1. Masses recorded for the twelve individual sulfonation and carbonization experiments. For each replicate set, about 20 g of pristine PP was divided into four portions of approximately 5 g; every portion was sulfonated independently and then carbonized at one temperature, so that each row is a separate experiment. m(PP) is the pristine PP charged, m(precursor) the washed and vacuum-dried sulfonated polymer, and m(carbon) the carbon powder recovered after carbonization and before ball milling. Masses were measured to 0.01 g.

**Replicate set**	**Sample**	**m(PP) (g)**	**m(precursor) (g)**	**Mass gain (wt%)**	**m(carbon) (g)**	**Yield vs precursor (wt%)**	**Yield vs pristine PP (wt%)**
1	PP-700	5.12	5.22	2.0	1.25	23.9	24.4
1	PP-800	5.02	5.14	2.4	1.20	23.3	23.9
1	PP-900	5.10	5.21	2.2	1.15	22.1	22.5
1	PP-1000	5.02	5.18	3.2	1.04	20.1	20.7
2	PP-700	5.13	5.36	4.5	1.24	23.1	24.2
2	PP-800	5.04	5.16	2.4	1.21	23.4	24.0
2	PP-900	5.11	5.23	2.3	1.17	22.4	22.9
2	PP-1000	5.09	5.33	4.7	1.12	21.0	22.0
3	PP-700	5.04	5.19	3.0	1.22	23.5	24.2
3	PP-800	5.11	5.18	1.4	1.21	23.4	23.7
3	PP-900	5.02	5.15	2.6	1.14	22.1	22.7
3	PP-1000	5.08	5.19	2.2	1.12	21.6	22.0

Table S2. Sulfonation mass gain, carbonization yield and overall yield for each carbonization temperature, as mean and standard deviation over the three independent replicate syntheses in Table S1. The carbonization yield is referred to the mass of the dried sulfonated precursor and the overall yield to the mass of the pristine PP charged; the two differ by the mass gained during sulfonation.

**Sample**	**Mass gain (wt%)**	**Yield vs precursor (wt%)**	**Yield vs pristine PP (wt%)**	**n**
PP-700	3.1 ± 1.3	23.5 ± 0.4	24.3 ± 0.1	3
PP-800	2.0 ± 0.6	23.4 ± 0.1	23.9 ± 0.2	3
PP-900	2.4 ± 0.2	22.2 ± 0.2	22.7 ± 0.2	3
PP-1000	3.4 ± 1.3	20.9 ± 0.8	21.6 ± 0.8	3

Figure S1

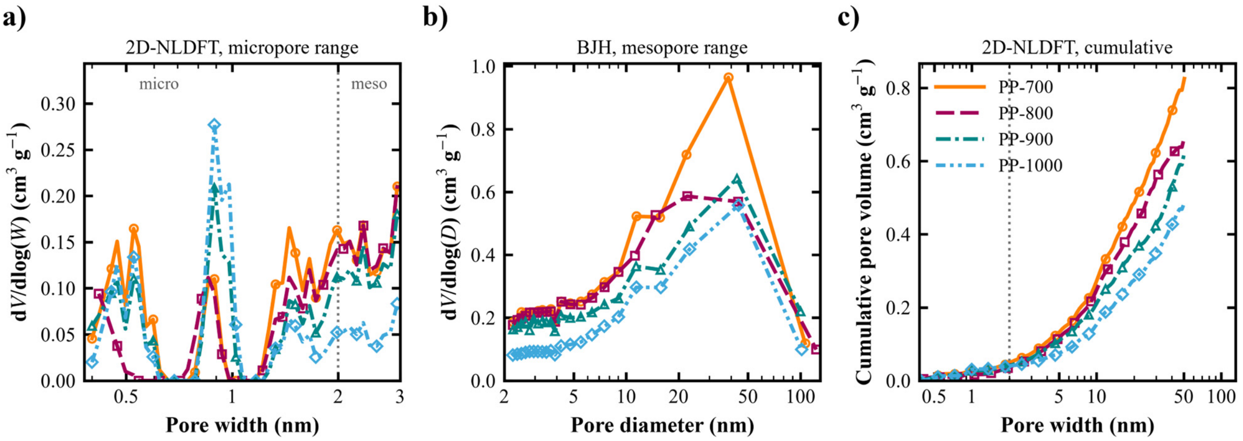

Figure S1. Pore size distributions of the PP-derived carbons from the nitrogen isotherms measured at 77 K. (a) 2D-NLDFT distribution over the micropore range. (b) BJH distribution over the mesopore range. (c) Cumulative pore volume from the 2D-NLDFT analysis. The dotted line marks the 2 nm boundary between micropores and mesopores. Each method is shown over the range in which it applies. Cumulative pore volumes from the two methods agree to within 3% for every sample.

Figure S2

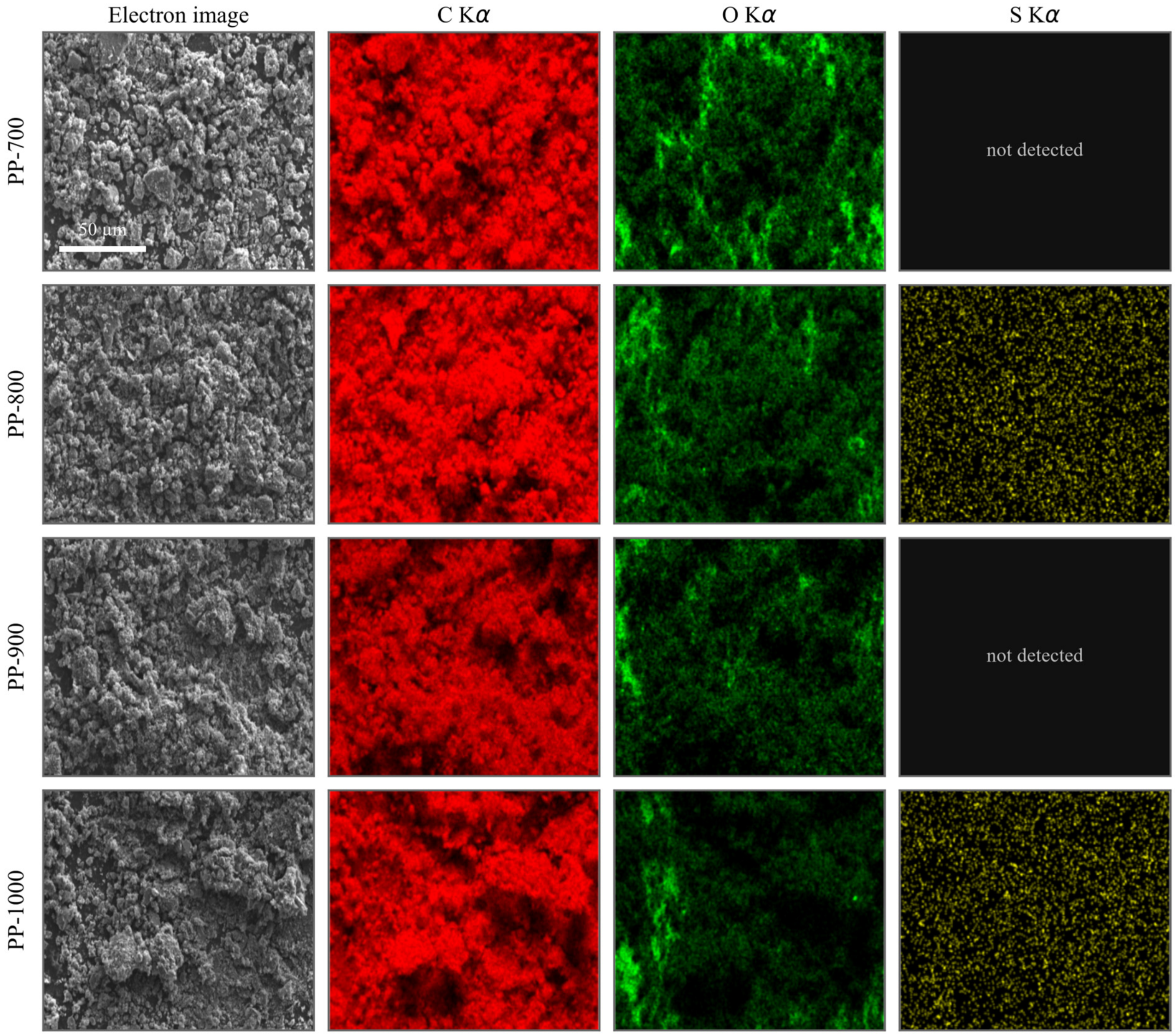

Figure S2. EDS elemental maps of the PP-derived carbons. Columns, from left to right: electron image, C Kα, O Kα and S Kα. Scale bar 50 µm, the same for every panel. Sulfur was below the detection limit in PP-700 and PP-900, so no sulfur map was recorded for those samples. Quantification from the map sum spectra: PP-700, 94.9 wt% C and 5.1 wt% O; PP-800, 95.3 wt% C, 4.5 wt% O and 0.2 wt% S; PP-900, 96.3 wt% C and 3.7 wt% O; PP-1000, 95.9 wt% C, 3.9 wt% O and 0.2 wt% S.
